# Vitamin K and its analogs: Potential avenues for prostate cancer management

**DOI:** 10.18632/oncotarget.17997

**Published:** 2017-05-19

**Authors:** Subramanyam Dasari, Syed M. Ali, Guoxing Zheng, Aoshuang Chen, Venkata Satish Dontaraju, Maarten C. Bosland, Andre Kajdacsy-Balla, Gnanasekar Munirathinam

**Affiliations:** ^1^ Department of Biomedical Sciences, College of Medicine, University of Illinois, Rockford, IL, USA; ^2^ Internal Medicine, Rockford Memorial Hospital, Rockford, IL, USA; ^3^ Department of Pathology, University of Illinois at Chicago, Chicago, IL, USA

**Keywords:** prostate cancer, Vitamin K, dietary constituents, apoptosis and autophagy

## Abstract

Epidemiological studies have demonstrated a relationship between cancer incidence and dietary habits. Especially intake of certain essential nutrients like vitamins has been shown to be beneficial in experimental studies and some clinical trials. Vitamin K (VK) is an essential nutrient involved in the blood clotting cascade, and there are considerable experimental data demonstrating its potential anticancer activity in several cancer types including prostate cancer. Previous *in vitro* and *in vivo* studies have focused mainly on anti-oxidative effects as the underlying anticancer mechanism of VK. However, recent studies reveal that VK inhibits the growth of cancer cells through other mechanisms, including apoptosis, cell cycle arrest, autophagy, and modulation of various transcription factors such as Myc and Fos. In the present review, we focus on the anticancer effect of dietary VK and its analogs on prostate cancer, with an emphasis on the signaling pathways that are activated following exposure to these compounds. This review also highlights the potential of VK and its derivatives as an adjuvant treatment in combination with other vitamins or with chemotherapeutic drugs. Based on our recent results and a review of the existing literature, we present evidence that VK and its derivatives can potentially be explored as cancer therapy, especially for prostate cancer.

## INTRODUCTION

Prostate cancer (PCa) is the second most common non-skin malignancy in developed countries like USA. More than 8,900,000 cases are detected and more than 260,000 deaths recorded worldwide every year [1]. The high death rate from PCa may be due to its natural history in which early stage cancer eventually progress into hormone refractory or castration resistant stage [2]. Histopathological analysis of clinical samples and animal studies have shown that infectious agents, dietary carcinogens, race, age and other factors can cause damage to the epithelial cells of prostate and elicit inflammatory responses predisposing to PCa [3]. Well-established pathophysiological evidence indicates that these inflammatory responses can lead to mutations [4, 5]. These mutations, together with inflammatory mediators and enhanced cell proliferation, are some of the causative factors for initiation and development of PCa [1]. These factors cause the formation of preneoplastic lesions including proliferative inflammatory atrophy (PIA) and prostate intraepithelial neoplasia (PIN) which can progress to malignancy and eventually lead to metastases. Progression of PCa is dependent on circulating levels of androgen until androgen independent, castration-resistant, cancer develops.

In view of the high mortality rate of PCa, a range of treatments have been developed for treating PCa. In most cases, localized PCa can be successfully treated by surgery or radiotherapy. But this is typically unsuccessful and complex when the cancer has become advanced and metastatic. Although conventional androgen deprivation therapy (ADT) is used to initially treat advanced PCa, unfortunately this approach invariably leads to resistance and its clinical use is only temporarily effective [6]. Failure of ADT leads to the development of so-called castration resistant prostate cancer (CRPC) [7–9]. Treatment options available for patients with CRPC are limited and more complicated than those for hormone-dependent PCa. Most chemotherapeutic drugs have inherent limitations such as development of resistance and side effects such as urinary dysfunction, bowel dysfunction and heart related complications. Therefore, there is an urgent need for the development of novel therapeutic approaches as well as chemopreventive strategies that can delay or reduce the progression of PCa with minimal side effects [10, 11]. This may be achieved through the identification and development of chemopreventive compounds from non-toxic natural sources such as dietary constituents.

### Role of dietary constituents in chemoprevention of PCa

Epidemiological studies have indicated that diet and nutrition are important determinants of PCa risk with significant variations in incidences between different geographic regions [12, 13]. Western nations in America and Europe have higher PCa incidence rates, when compared to many Asian countries [14]. The lower rate of PCa in Asian countries may be due to dietary patterns with low fat intake and diets rich in plant-based agents. Hence, the addition of vegetables and fruits in the daily diet to prevent PCa has received considerable attention for cancer chemoprevention [15].

Chemoprevention is defined as prevention of cancer development or inhibition of cancer progression through pharmacological intervention with drugs, naturally occurring compounds, or dietary supplements [15]. According to Ansari et al. [16, 17] and Montironi et al. [18], chemoprevention of cancer may be defined as inhibition or delay of the onset of cancer growth by blocking neoplastic transformation as well as reversing the progression of transformed cells to the malignant stage. Epidemiological studies have indicated that populations consuming vegetables rich in micronutrients (anti-oxidants, vitamins, and trace minerals) have lower cancer incidence and cancer mortality [19]. Further research studies suggested that micronutrients present in vegetables and fruits may have potent anticancer properties [20]. Hence, there is a considerable interest in ascertaining whether these micronutrients, including vitamins such as VK, offer protection against PCa [19, 20]. A number of macro- and micro-nutrients have been proposed as chemopreventive agents [21]. Dietary or natural compounds may exert their chemopreventive effects by a variety of mechanisms, for example, by scavenging oxygen free radicals or inhibiting polyamine metabolism, thereby preventing carcinogenesis [22, 23]. Natural compounds are also known to exhibit anticancer effects through regulation of signal transduction pathways, induction of apoptosis, and activation of anti-metastasis genes [24]. Some well-known dietary agents that have been studied as potential chemopreventives include micronutrients such as selenium, vitamins C, D, E, and K, lycopene, green tea, silymarin, pomegranate and resveratrol. These agents may exert their putative anticancer activity through various mechanisms as shown in Table 1 [14].

**Table 1:** Chemopreventive mechanism of dietary agents in prostate cancer

Dietary agents	Source of the dietary agent	Mechanism
Lycopene	Tomato	Influence expression of gap junction proteins and growth factor signaling [153]
Vitamin A	Fruits and vegetables	Apoptosis, cell cycle arrest [154]
Vitamin C	Fruits	Cellular chromosomal damage [37]
Vitamin D	Residential sunlight and milk with Vitamin D	Apoptosis, cell cycle arrest [42]
Vitamin E	Fruits and vegetables	Inhibition of protein kinase C, induction of NADPH detoxification enzyme and reduction of arachidonic acid metabolism [155]
VK2	Fruits, vegetables, meat	Apoptosis, ROS production [84–86]
Selenium	Fruits and vegetables	Cell cycle arrest and apoptosis and reduction of angiogenesis [156]
Selenium + Vitamin E	Fruits and vegetables	Apoptosis, cell cycle arrest [157]
Soy flavonoid (genistein)	Soy seeds	Apoptosis and cell cycle arrest [158]
Tea (catechins)	Tea leaves	Cell cycle arrest, inhibition of angiogenesis, inhibition of protein kinase C and apoptosis [159]
Resveratrol	Peanuts, pistachios, grapes, blueberries, cranberries	Cell cycle arrest and apoptosis [160]
Silymarin	Medicinal plant	Cell cycle arrest, inhibition of mitogenic cell survival signaling [161]
Proanthocyanidins and procynadins	Grape seed extract	Inhibition of protein tyrosine kinase, matrix metalloproteinases and Rel/NF-kB family members [162]
Apigenin	Fruits and vegetables	Cell cycle deregulation and apoptosis [163]
Indoles and their derivatives	Fruits and vegetables	Modulations in cell cycle regulatory proteins and inhibition of cell survival pathways (PI3K/Akt) and NF-κB transcription factor [164]
Isothiocyanates	Cruciferous vegetables	Cell cycle inhibition, induction of phase II enzymes, inhibition of extracellular signal-regulated kinases, suppression of NF-κB [165]
Phenolic acids Curcumin	Component of turmeric	Tyrosine kinase and protein kinase C inhibition, down-regulation of AR gene expression, inhibition of PI3K/Akt and NF-κB [166]

### Vitamins and PCa chemoprevention

As indicated above, there is a considerable interest in micro-nutrients and other dietary agents as potential chemopreventive agents against malignancies including PCa [21]. The antioxidant and non-antioxidant activities of various dietary agents in PCa prevention have been examined in several studies [25, 26]. Most studies have addressed dietary sources that are rich in phytochemicals such as carotenoids, vitamins, flavonoids, selenium, dietary fiber, glucosinolate, indoles and phenols [27]. These phytochemical constituents have complimentary or overlapping mode of actions including antioxidant activity, enzyme detoxification, inhibition of nitrosamines formation, alteration of hormone metabolism and have the ability to modulate the carcinogenic cellular events [28].

It has been hypothesized that the lycopene present in tomato is associated with prevention of PCa. For instance, Hwang and Bowen [29] suggested that a diet rich in tomatoes and tomato products containing lycopene is associated with reduction of PCa risk. Some case-control studies and a meta-analysis also reveal that tomato products may play a role in the prevention of PCa [30, 31]. One preclinical study of PCa revealed that the consumption of tomato powder (but not pure lycopene) prevented prostate carcinogenesis in a rat model, suggesting that tomato powder contain some anticancer compounds other than the lycopene that inhibits the carcinogenic process [32]. On the other hand, other animal studies and a recent phase II intervention study with a lycopene-rich tomato product did not yield results indicative of preventive efficacy against PCa [33].

A major aspect in PCa chemoprevention through dietary constituents has focused on micronutrients, especially vitamins such as vitamin A, C, D and E, which have been extensively studied for their effects on PCa.

Vitamin A is essential for cell differentiation, visual functioning, physiological growth and is known to be able to modulate the cancer cell growth [34]. The potential chemopreventive mechanisms of vitamin A and its analogues have been shown in laboratory studies to specifically act on the tumor progression stage through the inhibition of cell proliferation, induction of apoptosis, cell cycle arrest and also a combination of these mechanisms [35]. However, toxicity has prevented clinical translation of the use of vitamin A and other retinoids to prevent PCa.

Vitamin C is a potent antioxidant which scavenges reactive oxygen species (ROS) and free radicals that cause DNA damage [36]. One potential chemopreventive mechanism of vitamin C has been shown to be the inhibition of neoplastic transformation by reducing cellular chromosomal damage [37]. Vitamin C not only acts as antioxidant by scavenging free radicals, but also reinstates the activity of α-tocopherol following its lipid peroxidation chain breaking effect and inhibits the growth of PCa cells *in vitro* [26]. In addition, Taper et al. [38] demonstrated that vitamin C inhibited the growth of both androgen-dependent and -independent human PCa cells in nude mice. Apart from the antioxidant mechanism of vitamin C, the combination of vitamin C with amino acids and other micronutrients are also effective in targeting the signal transduction pathways to inhibit the cell proliferation and cancer progression in laboratory studies, as has been shown for ovarian cancer [39].

Vitamin D (calcitrol) is synthesized in the skin following exposure of 7-dehydrocholesterol to ultraviolet light and is derived from dietary sources. Exposure to residential sunlight is associated with decreased risk of PCa that may be linked with calcitrol synthesis [40]. In addition, some epidemiological studies have suggested that increased risk of PCa is associated with a decreased production of vitamin D [41]. The biologically active form of vitamin D inhibits PCa cell proliferation *in vitro* through various mechanisms including induction of apoptosis, cell cycle arrest, and activation of growth factor signaling [42]. The combination of vitamin D with other dietary constituents such as genistein (component of soy) has also been shown to inhibit the growth of benign primary human prostate epithelial cells and PCa cells [43]. Statistical analysis of PCa mortality rates in 71 countries showed that exposure to increased sunlight and consumption of oilseeds and soybeans was inversely correlated with the rate of PCa [44].

Vitamin E is a group of naturally occurring compounds: the tocopherols, tocotrienols and their derivatives. Of all the tocopherols, α-tocopherol is the predominant form of vitamin E found in plasma and tissues. Epidemiological studies have shown that consumption of a diet rich in vitamin E is inversely associated with the rate of PCa incidence [45, 46]. However, some epidemiological studies did not support an anticancer role of vitamin E in PCa [47, 48]. Due to their ability to trap reactive oxygen and nitrogen species (RONS), tocopherols are important biological antioxidants, and their cancer preventive activities have been extensively studied [49, 50]. Besides their anti-oxidant activity, vitamin E and its derivatives exert their anticancer effects through altered transforming growth factors-β and androgen receptor/prostate specific antigen (AR/PSA) signaling pathways and by regulating the cell cycle arrests at synthesis phase in PCa cell lines [51]. The mechanisms through which vitamin E inhibits cell proliferation include inhibition of protein kinase C activity, enzyme detoxification, induction of apoptosis, regulation of Fas levels in the membrane and cytoplasm and inhibition of matrix metallo-proteinases [52]. Male transgenic TRAMP (transgenic adenocarcinoma of the mouse prostate) mice fed with vitamin E succinate, selenium and lycopene supplemented diet had a significant reduction in PCa incidence [53]. Despite these promising studies indicating anti-PCa activity of vitamin E, α-tocopherol supplementation did not reduce, but slightly increased PCa risk in a large randomized clinical trial, SELECT (selenium and vitamin E cancer prevention trial) [54, 55].

Selenium is an essential component of several antioxidant enzymes such as glutathione peroxidase and oral supplementation with a baker's yeast grown on a selenium-rich medium reduced PCa risk a small randomized trial. Furthermore, it causes cell cycle arrest and apoptosis and inhibits angiogenesis [56]. However, in the large randomized clinical trial, SELECT, it did not prevent PCa and in subgroups increased risk slightly [55, 57].

### Vitamin K (VK) as an anticancer agent

VK is an essential micro nutrient, primarily associated with action in the coagulation cascade, and it also regulates bone metabolism through a mechanism involving gamma carboxylation of bone matrix proteins [58]. In addition to this activity, VK also had anticancer activity against human cancers such as hepatic, leukemia, lung, colonic, oral, breast and bladder cancers *in vitro* [59, 60]. Naturally occurring VK in the human diet is VK1 (phylloquinone) found in plants, particularly in green leafy vegetables where it is involved in photosynthesis. Mammalian intestinal bacteria convert VK1 into VK2 (menaquinone), the physiologically active form of VK. There are three synthetic forms of VK, VK3 (menadione), VK4, and VK5. VK3 is used as source of VK in pet food and as dietary supplement in some countries. VK1 and VK2 are non-toxic even at high doses, but large doses of VK3 are toxic capable of causing hemolytic anemia, liver toxicity, and allergic reactions.

Major anticancer efficacy of VK1 and VK2 is mediated by non-oxidative mechanisms, probably via transcription factors, but VK3 at higher concentrations works via reducing oxidative stress and arylation. Addition of catalase to the culture medium inhibits the *in vitro* effects of VK3 but not VK2 which suggested that the VK are a class of growth inhibitors that have a novel mechanism possibly involving carboxylation. [61, 62]. Pharmacological studies demonstrated that massive doses of VK2, up to more than 2.5 grams given per day, were safe and caused no enhancement of the toxicity of chemotherapy [63]. VK is a family of naturally occurring essential fat soluble compounds derived from 2-methyl-1,4-naphthoquinone that are structurally characterized by a common quinone naphthalene ring carrying two carbonyl moieties [58]. Interestingly, quinones are the functional unit in several cancer chemotherapeutic drugs such as doxorubicin, daunomycin, mitomycin C, and mitoxanthrone and the basic structure of VK is also a quinone (Naphthoquinone). This in part explains why VK has gained interest for the prevention and treatment of cancer [64].

Tamori et al. [65] reported that VK2 prevents hepatocarcinogenesis in patients with hepatic cirrhosis. In a meta-analysis of four randomized controlled studies of the preventive effect of VK analogues on the incidence of HCC recurrence after partial hepatectomy or local ablative therapy, indicated that VK and its derivatives prolonged disease-free survival [66, 67]. A recent meta-analysis conducted by Zhong et al. [68] reported that an analogue of VK2 may prevent the formation of secondary tumors in residual liver tissue following partial hepatectomy and increase the overall survival rate by inhibiting or activating certain signaling pathways (intrinsic apoptotic pathway and inhibition of nuclear factor kappa B activation) in HCC patients.

The antitumor activity of VK was first reported almost six decades ago [69]. The intravenous administration of the synthetic derivative of VK, menadione (VK3) increased the survival of inoperable bronchial carcinoma patients [58]. VK3 also synergistically inhibit cancer cell growth specifically in combination with vitamin C by activating oxidative stress and depleting cellular thiols [70]. Similarly, phylloquinones (VK1) and menaquinone (VK2) exert anti-proliferative activity by targeting transcription factors of proto-oncogenes such as c-myc, c-jun and c-fos, which result in cell cycle arrest and apoptosis [71]. Both phylloquinone (VK1) and menaquinone (VK2) have anticancer effects in various cancer cells including of the breast, stomach and liver [72, 73]. Menadione (VK3) was also shown to exhibit potent cytotoxicity against several cancers including oral, prostate, renal and breast cancer [74–77].

### Epidemiological studies of VK and PCa

**Figure 1:**
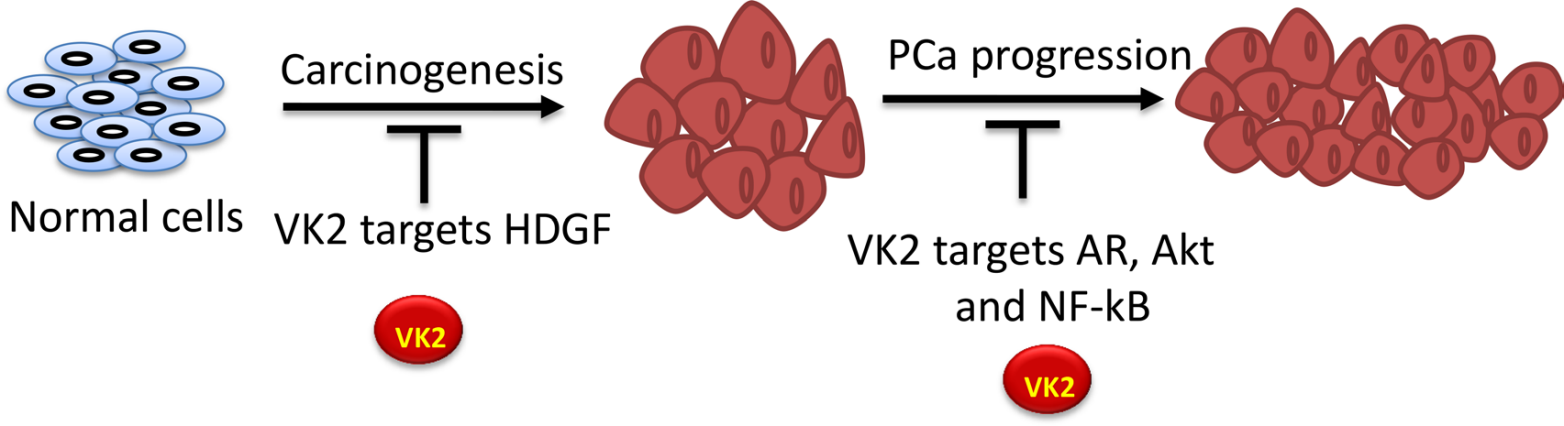
Proposed molecular mechanism of VK2 in prostate cancer VK2 targets HDGF protein during the development of carcinogenesis and targets androgen receptors (AR), Akt and NF-kB during the progression of PCa.

Epidemiological studies suggest that there is inverse association between dietary intake of VK (especially menaquinone) and overall cancer incidence [78]. This inverse association was seen in male patients with cancers of the prostate, colorectum, and lung, most profoundly for prostate and lung cancer [78]. A first confirmatory epidemiological study was reported by Nimptsch et al. [79] in which risk of advanced stage PCa was increased in participants with poor VK status as estimated by serum under-carboxylated osteocalcin. Nimptsch et al. [64] also conducted a cohort study in which they evaluated the association between dietary intake of VK (phylloquinone and menaquinone) and the risk of PCa. Their statistical analysis and experimental data indicated an inverse association of VK (menaquinone) and PCa risk. By contrast, such association was not observed for phylloquinone intake. In two other studies, short-term (6–12 weeks) and longtime treatment of VK were compared in their effect on urological cancers; the long-term VK treatment reduced risk of PCa by 40% more than the short-term VK treatment [80, 81]. By contrast, a small case–control study conducted by Blumentals et al. [17] suggested that there is no association between VK treatment and bladder cancer risk. Pottegard et al. [82] also reported no apparent association between intake of VK and total cancer risk in a population-based case control study. However, we recently reported that the administration of VK2 significantly inhibited proliferation of both androgen-dependent and androgen-independent PCa cells through molecular mechanisms involving induction of apoptosis and reduction of the angiogenic potential of the PCa cells [83]. Although the exact mode of action for the anticancer activity of VK2 is still unclear, some our above-mentioned study and other experimental studies suggest that VK2 has anticancer activity through the mechanisms such as induction of apoptosis, production of reactive oxygen species (ROS) and cell cycle arrest [84–86]. The anti-proliferative activity of VK2 has been most extensively studied in hepatocellular cancer in which ROS production, apoptosis and cell cycle arrest at S phase are implicated for its tumor suppressive role [82, 83]. Hepatoma-derived growth factor (HDGF) is a heparin- binding growth factor protein which was shown to be partially responsible for the anti-proliferative property of VK2 in hepatocellular cell lines [87]. Recently, we have reported that HDGF also plays an important role in the regulation of cell growth and apoptosis as well as invasion of human PCa cells, and hence it may serve as a therapeutic target for VK2 in PCa through the mechanism of the AKT and NF-kB pathways (Figure 1) [88]. Menadione (VK3), along with vitamin C (ascorbic acid), has been shown to reduce the rate of PCa growth in both *in vitro* and *in vivo* models [89]. The synergistic antitumor effects of VK3 and ascorbic acid appear to act through caspase-mediated apoptosis in PCa [74]. Recently, Gilloteaux et al. [90] reported that the combination of VK3 and ascorbic acid induces oxidative stress in DU-145 PCa cells. Scanning and transmission electron microscopy studies showed that the ROS induced by this combination damages nucleus, mitochondria, endomembranes, lysosomes, finally resulting in cell death [90].

Interestingly, pronounced anticancer effects of VK3 have also been observed when combined with other compounds such as plumbagin and juglone. Plumbagin (5-hydroxy-2-methyl-1,4-naphthoquinone) is naturally occurring napthoquinone sharing structural similarity with VK3 which is found in natural naphthoquinones such as roots, leaves, bark, and wood of Juglansregia (walnut). Zhou et al. [91] reported that plumbagin promotes the apoptosis and autophagy in PC-3 and DU145 PCa cells. They showed that plumbagin reduced the mitochondrial membrane potential followed by the release of cytochrome c in a dose-dependent manner which eventually led to apoptosis by the activation of the caspase cascade (caspase 9 and 3). They also reported that plumbagin induces autophagy by the inhibition of phosphatidylinositol 3-kinase (PI3K)/protein kinase B (Akt)/ mammalian target of rapamycin (mTOR) and p38 mitogen-activated protein kinase (MAPK) pathways and activation of 5′-AMP-dependent kinase (AMPK) in PC-3 and DU145 cells. Plumbagin also shows anticancer effects on BRCA 1/2 defective castrate resistant prostate cancer cells as well as prostate cancer stem-like cells by the activation of apoptosis [92]. Recently, Hafeez et al. [93] reported that the dietary plumbagin inhibits the growth of both primary and castration-resistant prostate cancer (CRPC) in Pten-knockout mice through the inhibition of PKC, Stat3, AKT and EMT markers (vimentin and slug), which are linked to the induction and progression of PCa. Collectively these studies suggest that VK and its derivatives hold promise both as a chemopreventive and as a therapeutic agent for PCa.

### Mechanisms underlying the anticancer effects of VK

All the three major analogues of VK (K1, K2 and K3) show anticancer activity such as induction of apoptosis and differentiation and cell cycle inhibition. Various anticancer mechanisms of VK are reviewed below.

### Oxidative stress-mediated anticancer effects of VK

Oxidative stress-mediated anticancer activity is believed to be the primary mode of action of VK. Oxidative stress is induced via redox-cycling of the Quinone to generate ROS such as hydroxyl, superoxide radical, and hydrogen peroxide [58]. If both redox-cycling and ROS surpass the anti-oxidative capacity of the cell, cell death results. ROS are chemically reactive molecules containing oxygen molecules produced by eukaryotic cells during normal oxidative metabolism through various mechanisms. An imbalance between the intracellular production of ROS and their defense mechanism leads to the oxidative stress. Augmented levels of oxidative stress induce lipid and protein oxidative modifications and DNA damage leading to apoptotic cell death or carcinogenic cell transformation [94]. Therefore, oxidative stress is involved in most of the pathological conditions and diseases including cancer, which is characterized by increased cell proliferation, accumulating mutations or other DNA damage, and genomic instability [95, 96]. It is well documented that cancer cells contain higher level of ROS than normal cells. Hence, cancer cells are more susceptible to oxidative stress-induced cell death and this could be exploited for the development of therapeutic approaches. Some chemotherapeutic compounds (such as cisplatin, Buthionine sulphoximine, and Imexon) specifically increase ROS production or inhibit ROS elimination by targeting scavenging systems, which induces cell death by the accumulation of ROS in cancer cells [97, 98].

**Figure 2:**
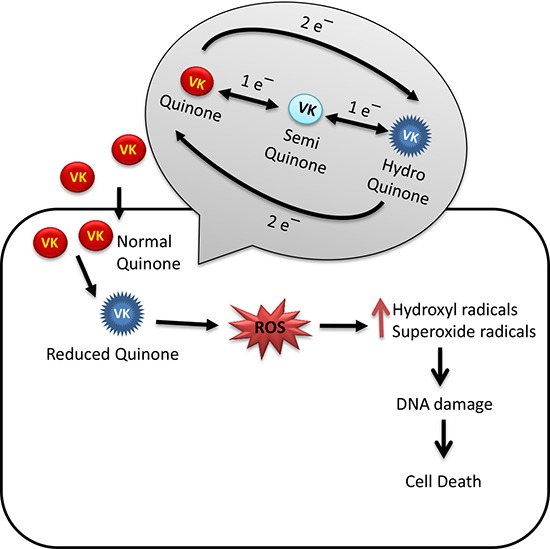
Oxidative stress mediated anticancer mechanism of VK VK undergoes redox cycling to form hydroquinone and generate reactive oxygen species (ROS). ROS mediated oxidative stress causes DNA damage and finally cytotoxic mediated cell death in cancer cells.

VK is a redox cycling compound that undergoes reduction producing semiquinone (one electron) followed by hydroquinone (two electrons) in cellular systems. Reduction of (one electron) VK is catalyzed by NADPH: cytochrome p450 reductase. In the presence of oxygen (O_2_), semiquinone is oxidized back to VK, which leads to reduction of O_2_ in the form of ROS. On the other hand, NQO1 uses NADH or NADPH as electron donors and catalyzes reduction of (2 electrons) VK (menaquinone) to hydroquinone in which reaction there is no generation of semiquinone and ROS production. The cyclic conversion of VK to semiquinone and back to quinone can result in the generation of ROS with adverse effects to the target cells. Oxidative stress increases DNA damage due to hydroxyl radicals produced by VK2 which leads to leads to cytotoxicity-mediated cell death in cancer cells (Figure 2) [99, 100].

### Apoptosis inducing potential of VK and targeting of apoptotic pathways

Apoptosis is characterized by specific morphological changes such as nuclear condensation and fragmentation, membrane blabbing and formation of apoptotic bodies [101]. VK has been shown to induce apoptosis through functional activation of Fas/FasL signaling in Jurkat cells [102]. Several external stimuli such as T-cell receptor (TCR) ligation, UV and gamma irradiation and drugs are able to induce apoptosis by upregulating FasL through the activation of transcription factors like nuclear factor of activating T-cells and NF-kB [102, 103]. Ligation of the TCR induces the expression of FasL which is dependent on c-myc expression. c-Myc may function as transcription factors to induce apoptosis when deprived growth factors and this c-myc-induced cell death may be mediated by cell surface interaction of Fas with its ligand (FasL) [104, 105].

Apoptosis is defined as a synchronized mode of cell death, in which an intracellular events act to eliminate the unwanted or dangerous cells [106]. Apoptosis has a wide range of biological significance playing key role in physiological processes like homoeostasis, differentiation, regulation and immune functioning [107, 108]. Homeostasis is maintained by a strict balance between apoptosis and cell proliferation. Disruption between this balance implicated in tumor development, neurodegeneration and autoimmune diseases [106, 109]. It is well established that many tumor promoters inhibits apoptosis, leads to development of chemo resistant tumors [110]. Activation of apoptosis in chemo resistant tumors is one of the challenging strategies in cancer treatment [111, 112]. Since alterations in apoptosis program can lead to inappropriate changes in apoptotic proteins that are altered to blunt the effects of drug treatment. Recently, VK has been extensively studied and found to exhibit a wide range of cytotoxicity against various cancer types both *in vitro* and *in vivo* through the induction of various apoptotic pathways as discussed below and summarized in Table 2.

**Table 2:** Distinctive mechanisms of different forms of VK that triggers programmed cell death by apoptosis

Different forms of Vitamin K	Cancer/Cancer Cell	Target molecule
VK	Pancreatic cancer	Caspase dependent apoptosis via the MAP kinase pathway [127]
VK2	Leukemia Leukemia and hepatocellular carcinoma	G0/G1 arrest along with Apoptosis [116] BaK mediated apoptosis [118] Regulating MMP [120] JNK cascade and FasL [126]
VK3	Leukemia	Fas/FasL [102]
VK3 + Vitamin C	Leukemia	ROS, NF-kB [113]
VK3 + Vitamin D-Fraction	Renal cell carcinoma	G0/G1 arrest along with Apoptosis [75]

The aforementioned synergistic effect of VK3 with vitamin C also involves induction of apoptotic cell death in leukemia cells by sequential molecular events involving the activation of NF-kB, ROS production, p53/c-Jun transcription factor, mitochondrial depolarization and caspase-3 activation pathway [87]. Other studies also indicated that the combination of VK and vitamin C induces apoptosis in leukemia cells by oxidative stress [113–115]. Miyazawa et al. [116] reported that VK2 causes G0/G1 arrest along with apoptosis induction especially in leukemia cells that are resistant to VK1 inducing apoptosis.

### VK targeting of cancer cells by mitochondrial mediated apoptosis

It is well established that mitochondria are an important component of the apoptosis execution pathway mediated by downregulation of Bcl-2 or Bcl-xl and mitochondrial membrane associated genes Bax and/or Bak [117, 118]. The anti-apoptotic Bcl-2 proteins regulates the mitochondrial membrane potential (MMP) and subsequent release of pro-apoptotic proteins such as cytochrome-c and apoptosis inducing factors (AIF) [117]. Cytoplasmic cytochrome-c interacts with apoptotic protease activating factor-1 (Apaf-1), leads to the activation of caspase family proteins which in turn leads to apoptosis through degradation of cellular proteins [119]. VK2 induces pro-apoptosis effects by regulating the MMP, in which mechanism VK2 produces superoxide within the mitochondrial membrane, followed by the release cytochrome c, activation of procaspase 3, and finally apoptotic cell death as shown in in TYK-nu ovarian cancer cells [120].

Yokoyama et al. [119] reported that VK2 induces apoptosis through mitochondrial caspase 3 mediated pathway in human myeloma cells and HL60 cells. In addition, Karasawa et al. [118] demonstrated the role of VK2 induced apoptosis through the activation of apoptotic regulators Bax and Bak in HL60 human promyelocytic leukemia cells. In this study, VK2-induced apoptosis was abrogated by the knockdown of Bak gene. Interestingly, VK2 directly binds to Bak, which is suppressed by anti-apoptotic Bcl-2 protein (Bcl-2 and Bcl-xL). Thus, the Bak gene is necessary for VK2-induced apoptosis and is therefore the molecular target of VK2. Furthermore, VK2 disrupted the mitochondrial membrane potential followed by the release of cytochrome C from mitochondria in a dose-dependent manner. Korsmeyer et al. [121] also reported that the loss of mitochondrial membrane potential and release of cytochrome C were associated with Bak and Bax in HL60 cells. Karasawa et al. [118] examined these pro-apoptotic proteins in human cervical carcinoma (HeLa) cells by immunofluorescent microscopy and found that VK2 induces activation and oligomerization of Bak and Bax. These results support the notion that VK2 induces mitochondrion-mediated apoptosis through the activation of Bak and Bax.

### Fas/FasL mediated apoptosis

**Figure 3:**
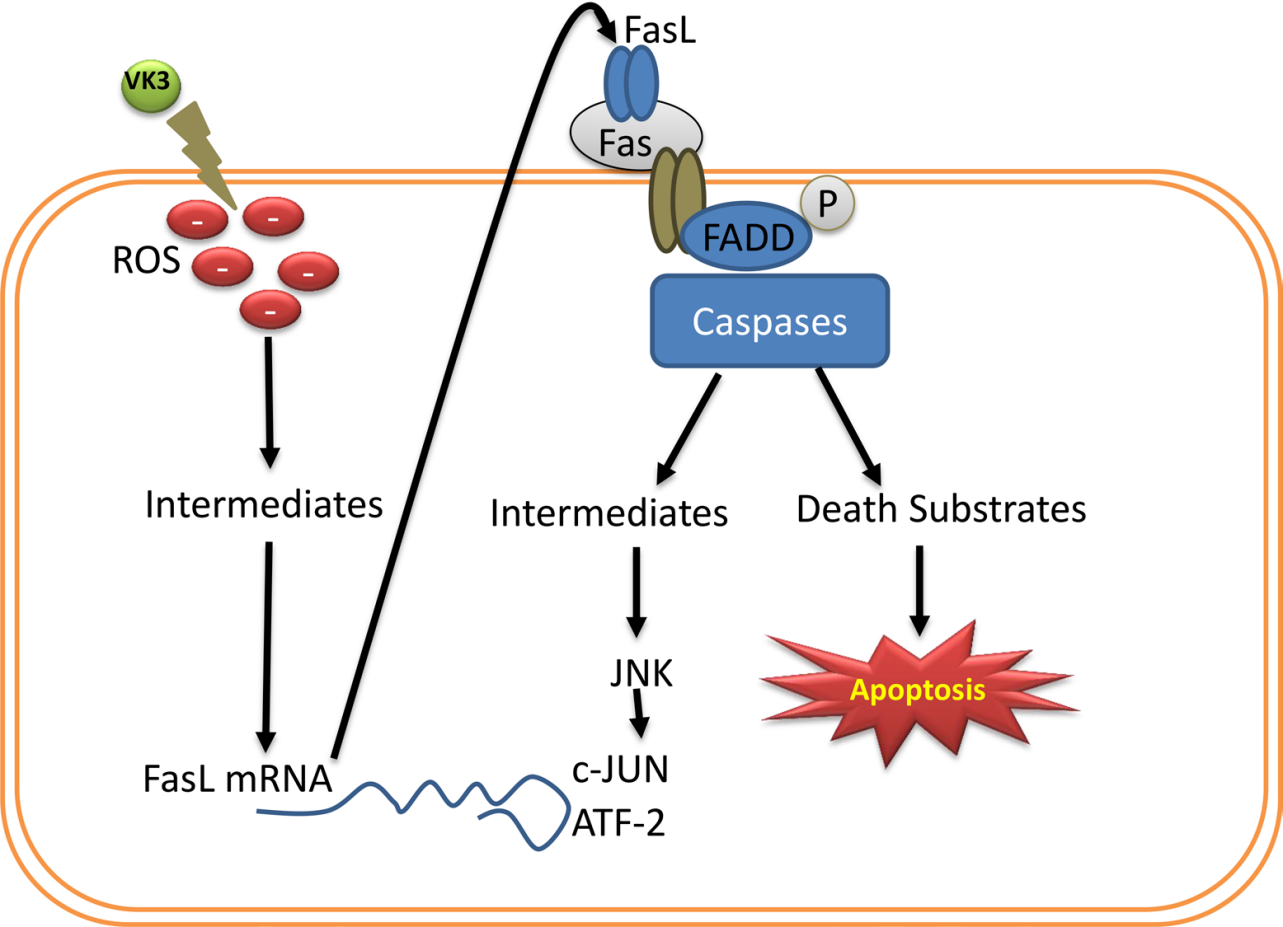
Regulation and expression of FasL during VK3 induced ROS VK induced ROS involved in Fas mediated apoptosis, intermediates of ROS regulate the expression of FasL mRNA, which in turn induces the caspase mediated apoptosis through phosphorylation of Fas associated protein with death domain (FADD).

Several apoptotic inducers such as certain chemotherapeutic drugs, UV and gamma irradiation, and TCR ligation are thought to induce apoptosis through the inactivation of transcription factors such as NF-kB [122]. Moreover, in T-lymphocytes, oxidative stress mediated apoptosis has been linked to upregulation of FasL through the activation of members of the stress-mediated protein kinase family [123]. c-Myc may function as a transcription factor to drive apoptosis when cells are deprived of growth or survival factors [104]. When amounts of survival factors are low, Fas interacts with its ligand (FasL) to induce c-myc mediated apoptotic cell death [124]. Caricchio et al. [102] found evidence that there is a direct association between c-myc and the Fas/FasL system in the effect of VK, as VK3 treatment induced c-myc and also increased both FasL and Fas. Another important property of VK3 is that it induces oxidative stress through the production of ROS. Oxidative stress is also involved in Fas-mediated apoptosis, in which reactive oxygen intermediates induce FasL mRNA expression and the antioxidant glutathione rapidly decreases during apoptosis induced by crosslinking of Fas receptors [102, 125]. Laux and Nel [126] reported that the JNK (Jun amino-terminal kinases) cascade and FasL expression are involved in VK3 (menadione) mediated apoptosis (Figure 3).

The mitochondrion is a major target for VK# (menadione)-induced cytotoxicity. Menadione induced ROS and thereby possible alteration of the oxidation-reduction state of cystenyl groups in the mitochondrial membrane. This, in turn, leads to the cross linking of sulphydryl (SH) groups in the permeability transition (PT) pore, leading to an increased/enlarged pore. PT pore opening initiates sequential events including decreasing mitochondrial membrane potential (Ψ) and disruption of inner membrane integrity. Ultimately it may result in the release of apoptotic proteins such as cyto-c, Apaf-1 which trigger caspase-9 activation. ROS intermediates induce expression of FasL protein which translocates from cytoplasm to plasma membrane to interact with Fas, which then recruits FADD (Fas-Associated protein with Death Domain) to bind to its death domain. This FasL-Fas activates the caspase cascade that subsequently leads to apoptosis and JNK-mediated cell death. Similar findings were observed in leukemia cells, in which VK2 induces apoptosis through the activation of caspase-3. It has been well established data that VK induces apoptosis in various cancer cell lines such as from renal cell carcinoma, leukemia, pancreatic cancer, glioma, and prostate cancer [75, 83, 116, 127, 128].

### VK2 induced autophagy

Similar to apoptosis, autophagy is an evolutionary conserved membrane mediated process that leads to the degradation of proteins and organelles present in cytosol by lysosomes [129]. Hence, autophagy is an important mechanism of living cells to remove damaged or long-lived cytosolic proteins. Autophagy-defective cells undergo susceptibility to metabolic stress and genomic damage, which finally may lead to tumorigenesis. Loss of the important autophagic gene *Beclin1* has been found in 75% of human breast, ovarian, and prostate cancers, suggesting that autophagy plays a critical role in preventing tumor cell growth. Paradoxically, autophagy has a dual role in cancer cells, functioning as both tumor suppressor by the removal of damaged proteins and organelles and promotion of tumor cell growth under adverse conditions due to stress tolerance [130]. In autophagy, cytosolic proteins and other organelles are engulfed into double membrane vesicles called autophagosomes which subsequently fuse with lysosomes to form autolysosomes to be degraded by lysosomal hydrolases [119]. Canonically autophagy is induced by nutrient deprivation and starvation and hence it is sensitive to levels of growth factors and hormones. Some cancer chemotherapeutic drugs also induce autophagy, such as a plant-derived alkaloid (voacamine), a Chinese herbal remedy (diterpenoidoritonin), and natural quinonoidplumbagin. Some autophagy inducing nutritional dietary constituents and their anticancer mechanisms are listed in Table 3 [131, 132]. Rapamycin is the currently available standard and potent drug to induce autophagy as therapy of various cancers [133]. Some trace elements and vitamins including vitamins C, D, and E have been shown to stimulate the autophagy in non-small cell lung cancer cells (vitamin C), head and neck squamous cell carcinoma cells (vitamin D_3_), and pancreatic cancer cells (vitamin E) [131]. VK2 also can induce autophagy as indicated by accumulation of autophagic vacuoles in cholangiocellular carcinoma cells treated with VK2 [134]. Other experimental studies indicated that VK2 inhibits the tumor cell growth by inducing both apoptosis and autophagy in leukemia and colon cancer cells [119].

**Table 3:** Induction of autophagy through nutritional and dietary constituents

Dietary Constituent	Cancer/Cancer Cell	Target molecule
Curcumin	Brain cancer	Akt/mTOR/S6 kinase, ERK1/2 [167]
Genistein	Ovarian cancer	Akt [168]
Resveratrol	Ovarian cancer Colorectal cancer Salivary gland cancer Lung cancer	Akt, mTOR, glycolysis [169, 170]
Sulforaphane	Prostate cancer	Mitochondria, mitophagy [171]
Vitamin C	Glial cells, Lung cancer	Not known [172, 173]
Vitamin D3	Head and Neck cancer	p19^INK4D^ [174]
VK2	Liver cancer	Not known
Ascorbate	Prostate cancer/PC-3 cells	ROS production [175]
Piperlongumine	PCa/PC-3 cells	ROS production and Akt/mTOR [176]
Curcumin	PCa/22Rv1	Prodeath [177]
Gossypol	PCa xenograft models	Prodeath [178]

### VK targeting of cancer cells by cell cycle arrest

Cell cycle checkpoint regulatory proteins (Cdk1, Cdk2 and Cdk4) are major effectors of regulation of the cell cycle. These check point regulators are coordinated by the interaction of various cyclins with their cyclin dependent kinases (CDKs) to form active complexes. Cell cycle check point failure often causes mutations and genetic instability which, in turn, can lead to the development of cancer [135]. Hence the identification of novel compounds that are capable of selective inhibition of these kinases (CDKs) are attractive strategies in cancer chemotherapy. Khan et al. [136] reported that pseudolaric acid B induces apoptosis through inhibition of CDKs activity, thereby causing cell cycle arrest, in glioblastoma cells.

**Figure 4:**
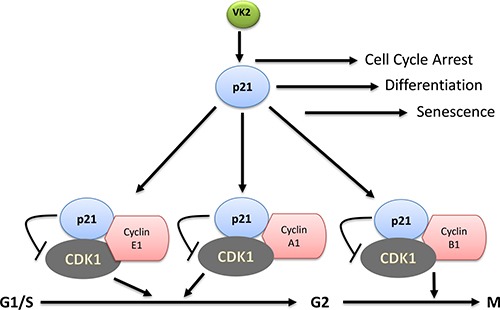
VK2 induced cell cycle regulation in cancer cells VK2 regulate the cell cycle arrest, cell differentiation and cellular senescence through cdk-1 inhibitor, p21. Subsequently, p21 inhibits the activity of cdk1 by binding with cyclin A1 and E1 and arrest the cell cycle at G1/S-G2 phase. Similarly, p21 inhibits activity of cdk1 by binding with cyclin B1 and arrest the growth at G2/M phase.

One of the important activities of VK3 is inducing cell cycle arrest by inhibiting the activity of CDK-1 check point as has been shown in cervical cancer cells [137]. VK3 causes hyper-phosphorylation on tyrosine residues on the epidermal growth factor receptor (EGFR) and cyclin dependent kinase-1 (CDK-1). Phosphorylation of CDK-1 itself is associated with reduced activity of both CDK-1 and protein tyrosine phosphates [138]. VK3 induces CDK-1 hyper-phosphorylation and also inhibit the cell division cycle 25A (CDC-25A) phosphatase activity, which is a dual specificity phosphatase in cell division and cell cycle, and finally leads to cycle arrest and cell death [137]. This is probably due to ability of VK3 to bind the catalytic domain of cdc25phophatase, thus interfering with the activity of CDC-25 phosphatase and delaying the cellular entry into the S and G2/M phase of cell cycle [137]. Cell division cycle 25 (CDC-25) is a protein tyrosine phosphate critical for cell division, especially at entry of S phase to G2/M phase. Miyazawa et al. [116] reported that VK2 stimulates one of the key CDK-1 inhibitors, p21, in a p53-independent manner. Generally, expression of p21 is upregulated by the p53 tumor suppressor gene, but p21 expression can also be regulated independently of p53 [116]. p21 inhibits cell cycle progression through the inhibition of CDK-2 activity, which is important for the phosphorylation of Rb and activation of E2F dependent gene expression. p21 is shown to uniquely function as a central inhibitor of cdk2 that is activated or induced in response to a variety of cellular signals to promote the tumor suppressor activities as shown in Figure 4.

### Analogues of VK

Some analogues of VK have potent anticancer activity. For example, Nishikawa et al. [139] demonstrated that synthetic quinoid analogues of VK have growth inhibitory effects on Hep3B hepatoma cells through both apoptotic and necrotic cell death and that these effects may be mediated by interaction at position 3 of their quinoid nuclei with cellular thiols. Tamura et al. [140] reported that the 2-(2-mercaptoethanol)-3-methyl-1,4- naphthoquinone analogue of VK had potent anticancer activity through the cell cycle arrest at both G_1_ and G_2_/M in hepatoma cells. The same analogue of VK was also shown to have more potent pro-apoptotic activity in primary rat liver cells via the mitogen-activated protein kinase pathway [141]. Kuo et al. [142] reported that plumbagin, an anologue of VK3, has anticancer effects by inhibiting cell proliferation by inducing cell cycle arrest at G2/M phase and autophagic cell death in breast cancer cells. Plumbagin may exert anticancer effects through various mechanisms including disruption of the microtubule network, production of ROS and cell cycle arrest [143]. Lee et al. [144] also reported that Plumbagin had potent antiproliferative activity through the intracellular production of ROS in breast cancer cells. In addition, Yan et al. [145] reported that Plumbagin can trigger DNA damage and also induce cell cycle arrest at S-phase in MG-63 human osteosarcoma cells. Furthermore, plumbagin has been shown to exhibit radiosensitizing effects both *in vitro* as well as in mouse models [146, 147].

### Role of VK as an adjuvant in cancer treatment

Cancer therapy modalities after surgery are either chemo-therapy or radiotherapy, sometimes in combination. Factors that can increase the effectiveness of cancer chemo- or radiotherapy are used as cancer adjuvant therapy. The combination of vitamins C and VK3 has been proposed as a non-toxic mixture of drugs active as an adjuvant cancer therapy by increasing chemo- or radiotherapy effects through alteration of deoxyribonuclease activity [148]. Gilloteaux et al. [149] reported that the co-administration of the non-toxic adjuvant Apatone (Vitamin C + VK3) to radiation and/or chemotherapy treatments to kill bladder and other cancer cells *in vitro* without any risk or side effects for patients. The combination of VK3 and ascorbate exhibited synergistic anticancer effect, associated with extracellular production of H_2_O_2_ that promoted cell death through DNA damage, lysosomal-mitochondrial perturbation and release of cytochrome c [150]. Several reports have indicated that combination of VK2 with certain cytotoxic drugs (sorafenib) exert synergistic anticancer effects [151, 152]. Sorafenib is an oral multikinase inhibitor and function as a single agent chemotherapeutic drug against hepatocellular carcinoma. Zhang et al. [151] investigated that the combination of both sorafenib and VK2 treatment strategy might be effective therapy against hepatocellular carcinoma than the treatment with sorafenib alone. Taken together, these studies support the potential of VK as an adjunct treatment for various cancers.

## CONCLUSIONS

In this review, we have summarized the recent progress of VK in various cancers especially PCa. Collective data from different studies indicate that VK is a potential anticancer compound. In particular, the following observations make VK a unique therapeutic agent for treatment of various cancers: (a) It exhibits a broad-spectrum of toxicity toward a wide range of human cancer cells of different origins; (b) It induces apoptosis by interfering with multiple mechanisms that are considered central to cancer development and progression; (c) It can inhibit multiple signaling pathways which are frequently deregulated in human cancers and associated with drug resistance.

Considering aforementioned outcomes, it can be speculated that VK and its derivatives and analogs may become potential compounds for future development of anticancer therapy. However, extensive preclinical and clinical trials are yet required to elucidate the full spectrum of anticancer effects of VK, either alone or in synergistic combination with other standard drugs, to validate its usefulness as a potent anticancer agent.
